# Follistatin288 Regulates Germ Cell Cyst Breakdown and Primordial Follicle Assembly in the Mouse Ovary

**DOI:** 10.1371/journal.pone.0129643

**Published:** 2015-06-15

**Authors:** Zhengpin Wang, Wanbao Niu, Yijing Wang, Zhen Teng, Jia Wen, Guoliang Xia, Chao Wang

**Affiliations:** State Key Laboratory of Agro-Biotechnology, College of Biological Sciences, China Agricultural University, Beijing, 100193, People’s Republic of China; Qingdao Agricultural University, CHINA

## Abstract

In mammals, the primordial follicle pool represents the entire reproductive potential of a female. The transforming growth factor-β (TGF-β) family member activin (ACT) contributes to folliculogenesis, although the exact mechanism is not known. The role of FST288, the strongest ACT-neutralizing isoform of follistatin (FST), during cyst breakdown and primordial follicle formation in the fetal mice ovary was assessed using an *in vitro* culture system. FST was continuously expressed in the oocytes as well as the cuboidal granulosa cells of growing follicles in perinatal mouse ovaries. Treatment with FST288 delayed germ cell nest breakdown, particularly near the periphery of the ovary, and dramatically decreased the percentage of primordial follicles. In addition, there was a dramatic decrease in proliferation of granulosa cells and somatic cell expression of Notch signaling was impaired. In conclusion, FST288 impacts germ cell nest breakdown and primordial follicle assembly by inhibiting somatic cell proliferation.

## Introduction

Mammalian female reproductive productivity and duration are determined by the primordial follicle pool, which represents nearly all available oocytes [1]. In mice, prior to primordial follicle formation, primordial germ cells undergo mitosis with incomplete cytokinesis and form germline nests or cysts from 10.5 days post coitus (dpc). At 13.5 dpc they start to undergo meiosis and then arrest at the meiotic diplotene stage at approximately 17.5 dpc [2]. The primordial follicles are assembled when the germ cell cysts breakdown and pre-granulosa cells invade at around 19.5 dpc and this continues until the third day post parturition (dpp) [3]. It is well known that most of the primordial follicles are developed by 6 dpp in mice [4–5]. Unfortunately, due to the high degree of complexity in the interactions between somatic cells and germ cells, the exact mechanism of primordial folliculogenesis remains unknown. Further studies are needed to elucidate the respective actions of both the oocytes and the pre-granulosa cells while the primordial follicles are developing and to identify molecules that modulate germ cell-somatic cell communication during folliculogenesis.

Several important pathways have recently been identified that may regulate the process of primordial follicle formation. For instance, in the neonatal mouse ovary, both estradiol and progesterone are critical for inhibiting nest breakdown and primordial follicle formation [6–7]. In addition, progesterone has been shown to prevent nest breakdown through down-regulation of Notch signaling [8]. Finally, transforming growth factor-β (TGF-β) family members, such as TGF-β, activin (ACT) and inhibin (INH) have multiple functions *in vivo* during early folliculogenesis [5, 9–12]. TGF-β ligands are either homodimeric or heterodimeric molecules that bind to serine/threonine kinase receptor types I and II on the cell surface to form a complex that activates the smad signaling pathway via a phosphorylation of smad proteins [11, 13–14]. We recently showed that TGF-β participates in the maintenance of the primordial follicle pool in the mouse ovary [10]. Also, ACT is produced by gonadal sources and stimulates follicle stimulating hormone (FSH) secretion in the pituitary [15]. Consecutive injection of ACT for 4 days into newborn female mice results in increased primordial follicle pool size, indicating that ACT contributes to folliculogenesis, although the exact mechanism is not known [5].

ACT can be bound and neutralized by follistatin (FST), a cysteine-rich monomeric glycoprotein [16–19]. Systematic depletion of *FST* in adult mice results in early lethality [20], while conditional deletion of *FST* in granulosa cells negatively impacts fertility by reducing the number of ovarian follicles and causing ovulation and fertilization defects [21]. Interestingly, FST is encoded by a single gene but is comprised of 3 isoforms that differ in the length of exon 6, located at the C terminus [16, 22]. Therefore, identifying the role of each individual isoform is vital to in order clarify how FST contributes to primordial follicle formation. Depletion of both *FST303* and *FST315*, two isoforms of FST, delays germ cell nest breakdown and reduces apoptosis in mice [23], indicating that both isoforms are involved in primordial folliculogenesis. However, the function of the strongest ACT-neutralizing isoform [24], FST288, on primordial follicle formation remains unclear. Therefore, in this study, we determined the role of FST288 during cyst breakdown and primordial follicle formation in the fetal mice ovary using an *in vitro* culture system.

## Materials and Methods

### Animals

Kunming white mice were purchased from the Laboratory Animal Centre of the Institute of Genetics (Beijing, China). They were maintained in the University Animal Care Facility under a light-dark cycle and had free access to food and water. Female mice (6 to 8 weeks old) were mated with adult male mice to induce pregnancy. Mice with a vaginal plug the next morning were considered to be at 0.5 dpc. To sacrifice the animals, pregnant mice were first anesthetized with isoflurane followed by cervical dislocation, mice pups were sacrificed by cervical dislocation on designated day. All efforts were made to minimize animal suffering. Experimental protocols were performed in accordance with institutional and national guidelines and regulations and were approved by the China Agricultural University Animal Care and Use Committee.

### Fetal ovary culture and chemicals

Ovaries were dissected from the mice as previously described[10, 25]. Fetal ovaries (17.5 dpc) were placed in pre-chilled PBS (10 mM, pH 7.4) and separated by microdissection from the mesonephros or ovarian capsule under a stereomicroscope. They were cultured in 6-well culture dishes (NEST Biotechnology, Beijing, China) in 1-mL basic medium DMEM/F-12 (GIBCO, Life Technologies, Carlsbad, CA, USA) at 37°C in 5% CO_2_ with saturated humidity. Ovaries were cultured in medium alone, in medium plus recombinant mouse Follistatin 288 (FST288) (R&D Systems, Minneapolis, MN, USA) at 500 ng/mL, or in medium plus recombinant human activin A (R&D Systems, Minneapolis, MN, USA) at 100 ng/mL for up to 7 days (equivalent to 5 dpp). The medium was supplemented with penicillin and streptomycin to prevent bacterial contamination and was changed every other day. 5-Bromo-2′-deoxyuridine (BrdU) is a thymidine analogue which can be incorporated into the newly synthesized DNA of replicating cells (during the S phase of the cell cycle), substituting for thymidine during DNA replication. BrdU is routinely and extensively used to measure DNA synthesis and to label dividing cells. For the 5-Bromo-2′-deoxyuridine (BrdU, Sigma-Aldrich, St. Louis, MO, USA) incorporation assay, BrdU was added to the cultured ovaries for 2 hours before the ovaries were collected and examined using immunohistochemistry with an anti-BrdU antibody as described below.

### Ovarian follicle counts

Ovarian germ cells and follicles were quantified as previously described [26–27]. In brief, ovaries were fixed in cold 4% paraformaldehyde for 24 hours, embedded in paraffin, and serially sectioned to a thickness of 5 μm. Sections were stained with hematoxylin and every fifth section was analyzed for the presence of germ cells and follicles. The follicles were categorized as follows: germ cell nest (unassembled oocytes grouped together with no intervening of pre-granulosa cells), primordial follicle (a single oocyte surrounded by several flattened pre-granulosa cells), and growing follicle (an enlarged oocyte surrounded by a mixture of squamous and cuboidal somatic cells or by one layer of cuboidal granulosa cells). Germ cell and follicle counts were multiplied by five to obtain cumulative counts for the entire ovary [27]. In order to quantify oocytes, the number of oocytes in two consecutive sections of the center of the ovary with the largest cross-sections was averaged. This method using two mid-diameter cross-sections has previously been shown to yield similar results as using all serial sections [26]. Only oocytes with a visible nucleus were counted, and 100–300 oocytes were present in each cross-section.

### Immunohistochemistry

Sections were de-waxed and rehydrated. Following antigen retrieval with 0.01% sodium citrate buffer (pH 6.0), the sections were immunostained with primary antibodies overnight at 4°C. The antibodies were diluted as follows: anti-FST antibody (Santa Cruz Biotechnology Inc., Santa Cruz, CA, USA) (which is recommended for detection of follistatin isoforms 1–3 of mouse origin) at 1:100 and anti-BrdU antibody (Abcam; Cambridge, UK) at 1:200. Subsequently, the sections were incubated with biotinylated secondary antibody (Zhongshan Company; Beijing, China) and avidin-biotin-peroxidase (Zhongshan Company) before being exposed to diaminobenzidine (DAB; Zhongshan Company) for 1 min and counterstained with hematoxylin. Non-immunized rabbit serum or mouse serum were used as controls.

### TUNEL staining

The degree of oocyte apoptosis was measured by terminal deoxynucleotidyl transferase-mediated deoxyuridine triphosphate nick-end labeling (TUNEL) assay using an In Situ Apoptosis Detection Kit (S7101; Millipore, Bedford, MD, USA). Paraffin-embedded slides were treated according to the manufacturer’s instructions. TUNEL-positive follicles were deemed to be undergoing apoptosis at the time of fixation.

### Western blot

Total protein was extracted from ovaries with a MEM-R kit (Pierce; Rockford, IL, USA) according to the manufacturer’s protocol. Protein concentrations were measured using a bicinchoninic acid (BCA) procedure (CellChip; BJ Biotechnology Co., Ltd; Beijing, China). Proteins were separated by 10% sodium dodecyl sulfate polyacrylamide gel electrophoresis (SDS-PAGE) and then electrophoretically transferred onto a Protran nitrocellulose membrane (Schleicher & Schuell; Dassel, Germany). After the transfer, membranes were incubated for 1 h in 5% bovine serum albumin (BSA) in Tris-buffered saline with Tween (TBST; 20 mM Tris-HCl, 150 mM NaCl, and 0.1% Tween 20; pH 7.6) at room temperature. Membranes were incubated overnight at 4°C with the appropriate primary antibody: anti-FST antibody (Santa Cruz Biotechnology) at 1:100, anti-Notch1 antibody (Santa Cruz Biotechnology) at 1:200, anti-NICD1 antibody (anti-Notch1 intracellular domain, Abcam; Cambridge, UK) at 1:200, or anti-Hey2 antibody (Abcam) at 1:200, anti-p-smad2 antibody (Cell Signaling Technologies, Danvers, MA, USA) at 1:500, anti-smad2 antibody (Cell Signaling Technologies) at 1:500. After a wash in TBST, the membranes were incubated for 1 h at room temperature with the appropriate secondary antibody in TBST. Finally, the membranes were visualized using a SuperSignal West Pico (enhanced chemiluminescence) detection system (Pierce Chemical Co., Rockford, IL, USA). Levels of β-actin were measured as an internal loading control.

### Statistical analysis

All results are expressed as mean ± standard error of the mean (SEM) from three independent experiments. Data were analyzed using an analysis of variance (ANOVA) with the StatView software (SAS Institute, Inc.; Cary, NC, USA). Values of p < 0.05 were considered statistically significant.

## Results

### FST is expressed in the fetal mouse ovary

**Fig 1:**
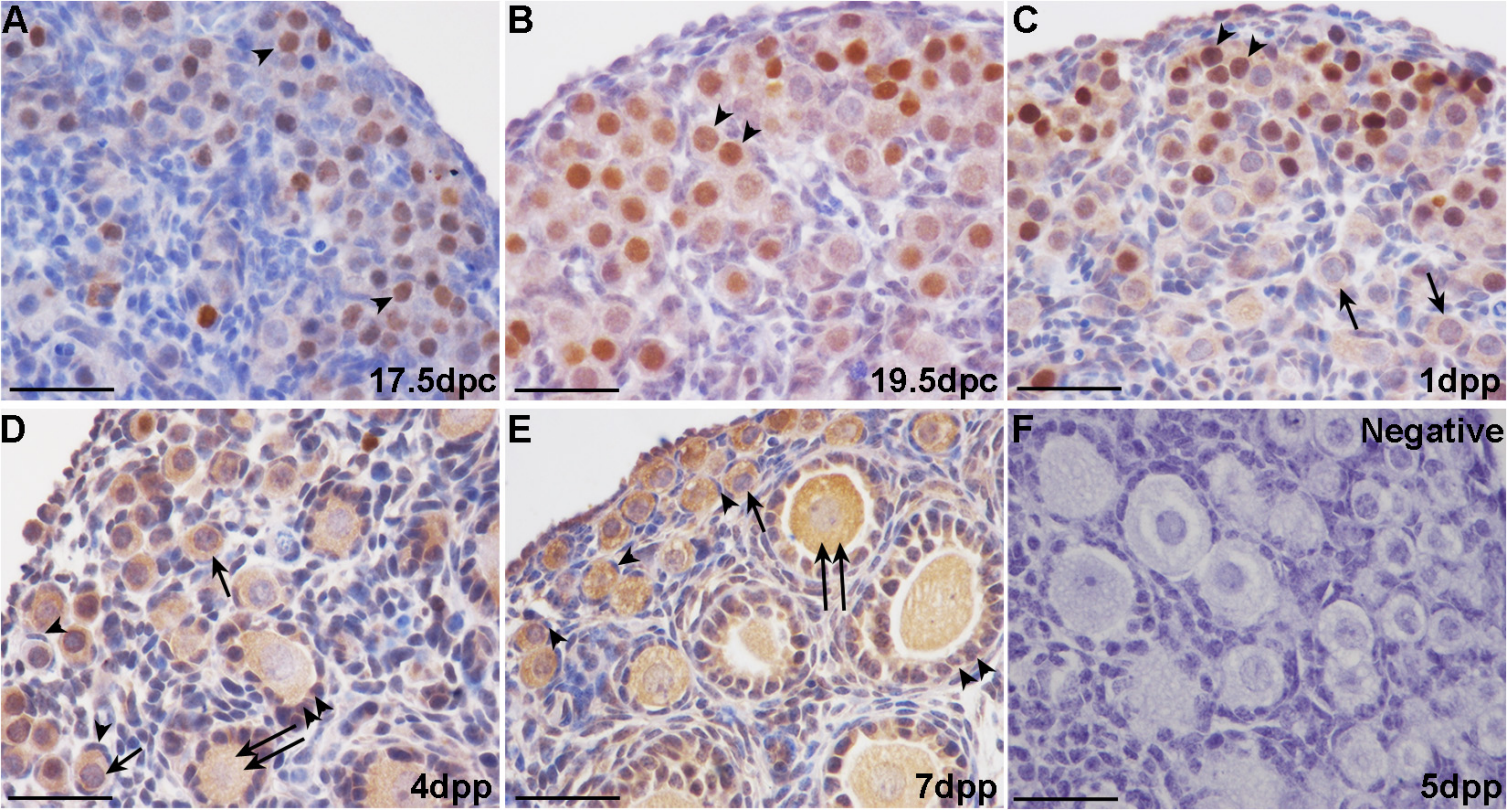
Immunohistochemical analysis of follistatin (FST) in prenatal and neonatal mouse ovaries. Weak FST staining was observed in the germ cell cytoplasm and strong staining in many germ cell nuclei (arrowheads) in 17.5 days post coitus (dpc) and 19.5 dpc mouse ovaries (A and B). FST continued to be expressed in the oocyte cytoplasm of nests and some primordial follicles (arrows) in 1 day post parturition (dpp) mouse ovaries; intense staining was observed in a portion of oocyte nuclei (arrowheads) (C). FST immunostaining in 4 and 7 days post parturition (dpp) mouse ovaries was prominent in the oocyte cytoplasm of primordial (arrows) and primary (double arrows) follicles as well as cuboidal granulosa cells (double arrowheads) of primary and growing follicles, but not in the flattened pre-granulosa cells (arrowheads) of primordial follicles (D and E). 5 dpp mouse ovaries were immunohistochemically stained with rabbit IgG as a negative control for FST (F). Scale bars: 40 μm (A-F).

**Fig 2:**
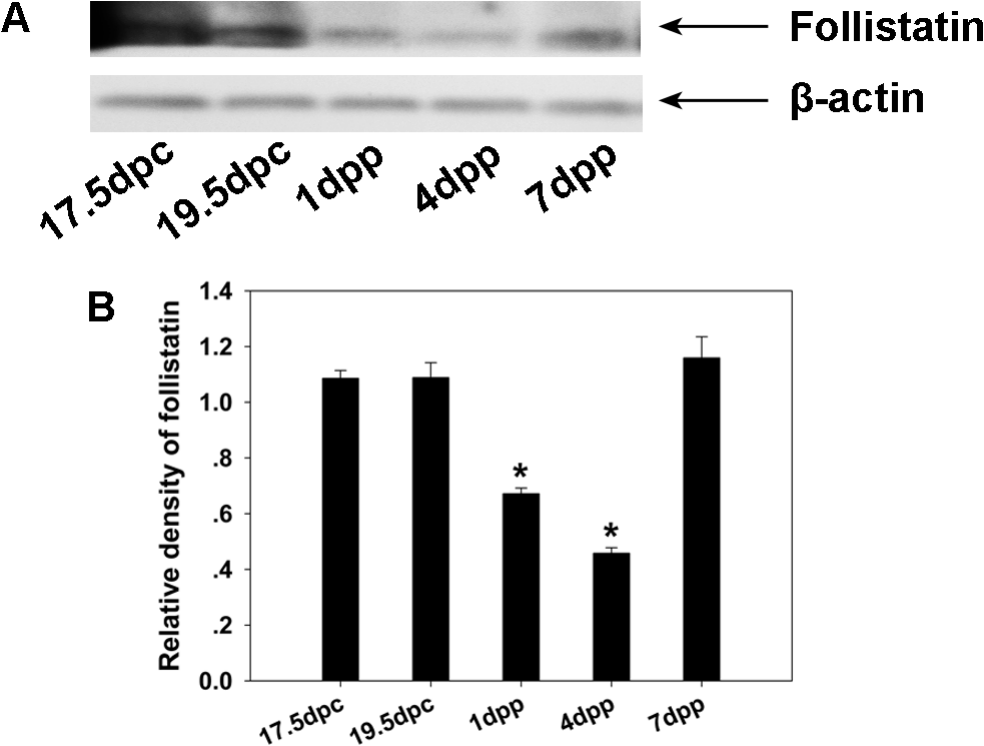
FST expression in the mouse ovary. Western blotting was performed to examine the mouse ovary FST protein levels from 17.5 dpc to 7 dpp, which were normalized to β-actin (A and B). Significant differences are indicated by * (*P* < 0.05).

Immunohistochemical and Western blot analyses were employed to determine the cellular localization and expression patterns of FST during germ cell nest breakdown and folliculogenesis. At 17.5 dpc and 19.5 dpc in mouse ovaries, FST staining is relatively weaker in the cell cytoplasm but stronger in the nuclei of many germ cells (Fig 1A and 1B). Somatic cells are FST-negative. At 1 dpp, FST is expressed in the oocyte cytoplasm of both nests and in some primordial follicles, with intense staining occurring in a portion of the oocyte nuclei (Fig 1C). By 4 dpp and 7 dpp, FST expression is prominent in the oocyte cytoplasm of primordial and primary follicles and in the cuboidal granulosa cells of primary and growing follicles, but not in the flattened pre-granulosa cells of primordial follicles (Fig 1D and 1E). Western blot results revealed that the FST protein is highly expressed in the ovary from 17.5 to 19.5 dpc, which is when the germ cells remain in nests. After birth, FST expression in the ovary decreases gradually from 1 dpp to 4 dpp while the primordial follicles assemble. FST expression declines to a nadir at 4 dpp and then recovers at 7 dpp (Fig 2A and 2B).

### Germ cell nest breakdown is delayed in FST288-treated ovaries

**Fig 3:**
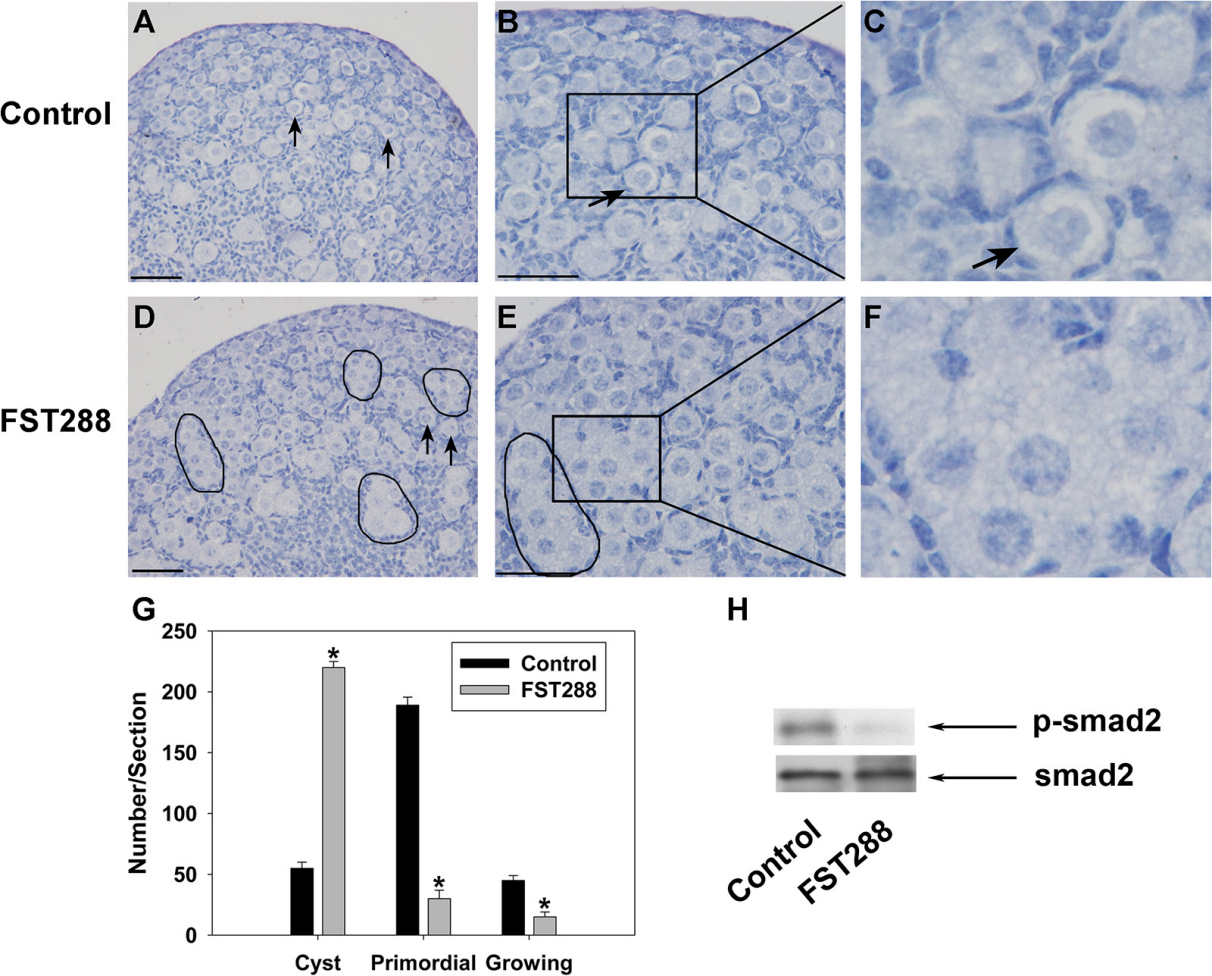
Phenotypes of ovary cultures treated with FST288. Ovaries at 17.5 dpc were cultured for 7 days without treatment (as a control) or with 500 ng/mL FST288. After culture, ovaries were fixed and sectioned, and then the shape and number of total germ cells and growing follicles were examined. Control ovaries had mostly primordial follicles containing small oocytes surrounded by flattened pre-granulosa cells (A and B, arrows, enlarged in C), while FST288-treated ovaries had a greater number of germ cells within nests (D and E, black boundaries, enlarged in F) compared to control ovaries. Follicle populations in the largest cross-section of control and FST288-treated ovaries were quantified (G). Western blot analysis revealed a dramatic decrease of the p-smad2 level in FST288-treated ovaries relative to control group (H). Scale bars: 40 μm (A, B, D and E). Smad2 was measured as internal control. Significant differences between control and FST288-treated ovaries are indicated by * (P < 0.05).

Ovaries (17.5 dpc) were cultured *in vitro* for 7 days (equivalent to 5 dpp) with or without 500 ng/mL FST288. The control ovaries were mostly composed of primordial follicles, with only a few small germ cell nests persisting near the ovarian cortex (Fig 3A–3C). In contrast, germ cell nest breakdown was delayed in FST288-treated ovaries, with a large number of germ cells remaining in nests, particularly near the periphery area of the ovary (Fig 3D–3F). Quantification of the total germ cells and growing follicles revealed that FST288-treated ovaries had a significantly higher number of germ cells that remained in nests and a significantly lower number of primordial and growing follicles compared to controls (Fig 3G). To check the effectiveness of FST288 on cultured ovaries, we measured the phosphorylation level of smad2 which could be activated by TGF-β superfamily members in the ovaries. Western blot analysis showed that the level of p-smad2 was dramatically decreased in FST288-treated ovaries as compared with control group (Fig 3H).

### Granulosa cell proliferation is arrested in FST288-treated ovaries

**Fig 4:**
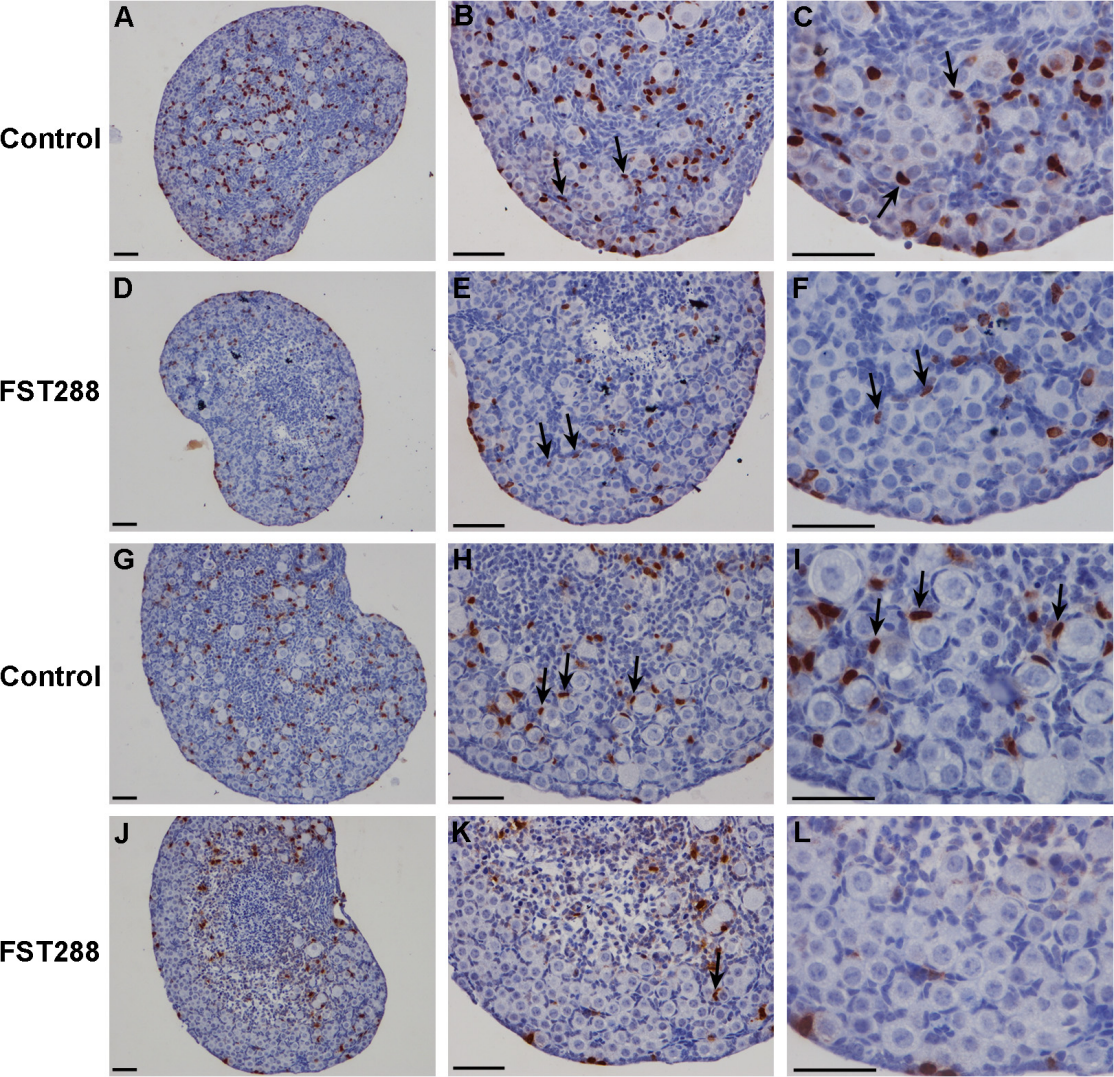
Proliferation of granulosa cells in FST288-treated ovaries. Ovaries at 17.5 dpc were cultured alone (as a control) or with 500 ng/mL FST288 for BrdU incorporation assays. After 4 days of culture, when compared to granulosa cells of control ovaries (A-C, arrows; BrdU-positive granulosa cells), BrdU incorporation into granulosa cells of FST288-treated ovaries was reduced (D-F, arrows; BrdU-positive granulosa cells), particularly near the ovarian cortex. Similarly, BrdU incorporation into granulosa cells near the periphery of FST288-treated ovaries (J-L) was reduced relative to granulosa cells of control ovaries after 7 days of culture (G-I, arrows; BrdU-positive granulosa cells). Almost all of the somatic cells near the ovarian cortex were BrdU-negative (J-L). Scale bars: 40 μm (A-L).

Mice ovaries (17.5 dpc) were cultured *in vitro* before granulosa cell counting. After 4 days of culture, BrdU incorporation assays revealed a dramatic decrease in granulosa cell proliferation near the ovarian cortex in FST288-treated ovaries compared to granulosa cells from control ovaries (Fig 4A–4F). Similarly, after 7 days of culture, very few granulosa cells were BrdU-positive in the FST288-treated ovaries, particularly near the periphery (Fig 4J–4L), while many granulosa cells had invaded into germ cell cysts to form primordial follicles in control ovaries (Fig 4G–4I). These results indicate an arrest of granulosa cell proliferation in FST288-treated ovaries.

### Germ cell apoptosis is unaffected in FST288-treated ovaries

**Fig 5:**
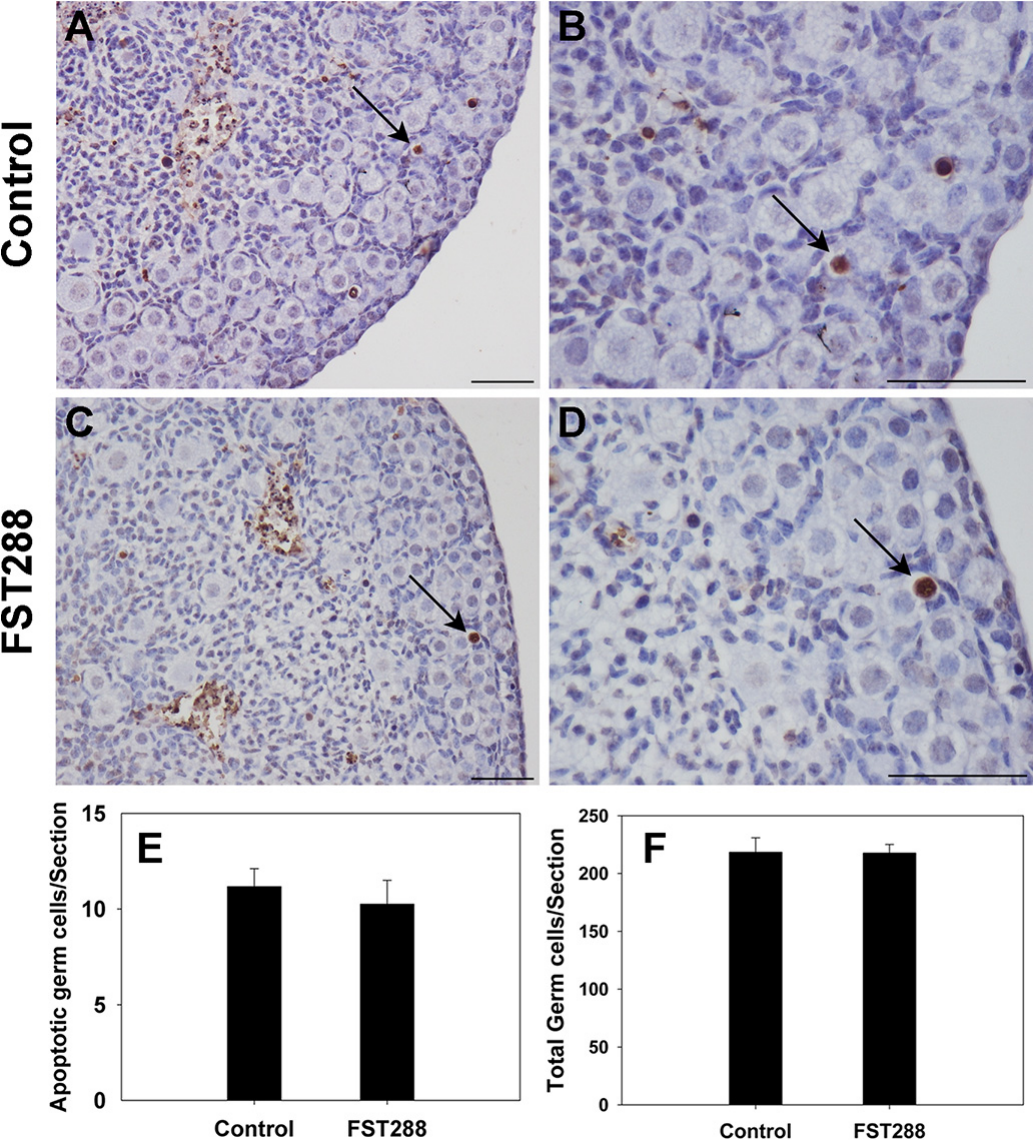
Germ cell apoptosis in FST288-treated mice ovaries. Ovaries at 17.5 dpc were cultured alone (as a control) or with 500 ng/mL FST288 for 4 days prior to apoptosis assay (TUNEL). After culture ovaries were fixed and sectioned, a TUNEL assay was performed on the sections. No significant difference in oocyte apoptosis was observed between control and FST288-treated ovaries (A-D, arrows; TUNEL-positive oocytes). Populations of apoptotic oocytes and total oocytes per section in each treatment group were quantified (E and F). Scale bars: 40 μm (A-D).

Since the primordial follicle assembly process involves programmed cell death of oocytes, a TUNEL assay for detecting apoptosis was performed on ovarian sections in the 17.5 dpc ovaries treated with 500 ng/mL FST288 for 4 days. Few TUNEL-positive oocytes were present in either control or FST288-treated ovaries (Fig 5A–5D). There was no significant difference in the number of apoptotic oocytes per section between control and FST288-treated ovaries (P > 0.05; Fig 5E). Also, the total number of oocytes was not significantly changed in each treatment group (P > 0.05; Fig 5F). Therefore, the actions of FST288 seemed not to alter oocyte apoptosis and oocyte number.

### Interaction between FST288 and notch signaling

**Fig 6:**
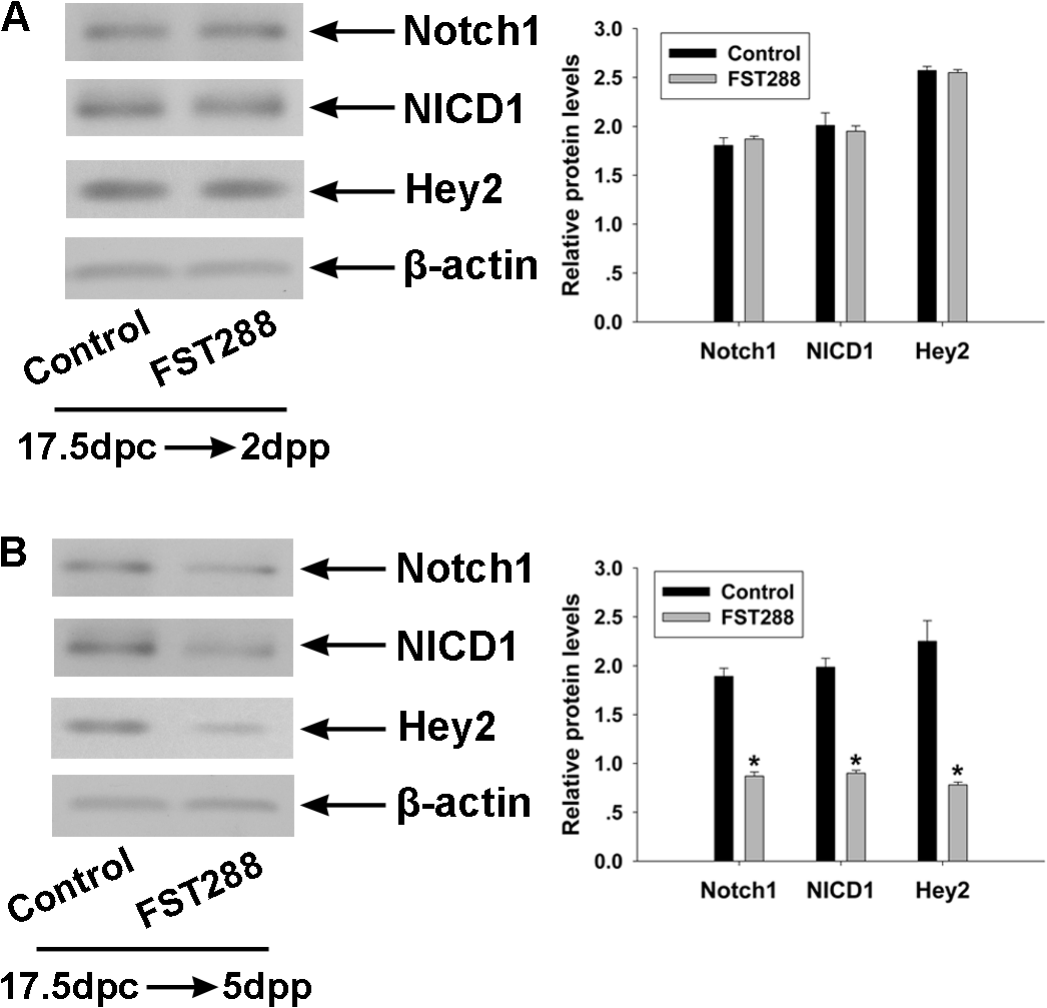
Effect of FST288 treatment on Notch signaling in ovaries cultured for 4 or 7 days. Ovaries at 17.5 dpc were cultured without treatment (as a control) or with 500 ng/mL FST288 for 4 or 7 days. The protein levels of Notch1, Notch1 intracellular domain (NICD1), and hairy/enhancer-of-split related with YRPW motif protein 2 (Hey2) were analyzed by Western blotting. Levels of Notch1, NICD1, and Hey2 were not altered in FST288-treated ovaries relative to control ovaries after 4 days of culture (A) and were significantly reduced after 7 days of culture (B). The protein level of β-actin was used as a loading control. Representative Western blot images are shown on the left and quantification of at least 3 independent Western blot results are shown on the right. Significant differences are indicated by * (P < 0.05).

Since Notch signaling can induce germ cell nest breakdown and primordial follicle formation, we investigated whether Notch signaling was affected in FST288-treated ovaries. Mice fetal ovaries at 17.5 dpc were cultured *in vitro* for 4 or 7 days following treatment with 500 ng/mL FST288, and then Western blot analyses were performed to detect the protein levels of Notch1, Notch1 intracellular domain (NICD1), and hairy/enhancer-of-split related with YRPW motif protein 2 (Hey2) which is a downstream responding protein of Notch. The levels of Notch1, NICD1, and Hey2 were unaffected at 4 days (Fig 6A), but at 7 days the protein levels were significantly decreased when compared to control (Fig 6B).

### Primordial follicle pool size is increased in ACT A-treated ovaries

**Fig 7:**
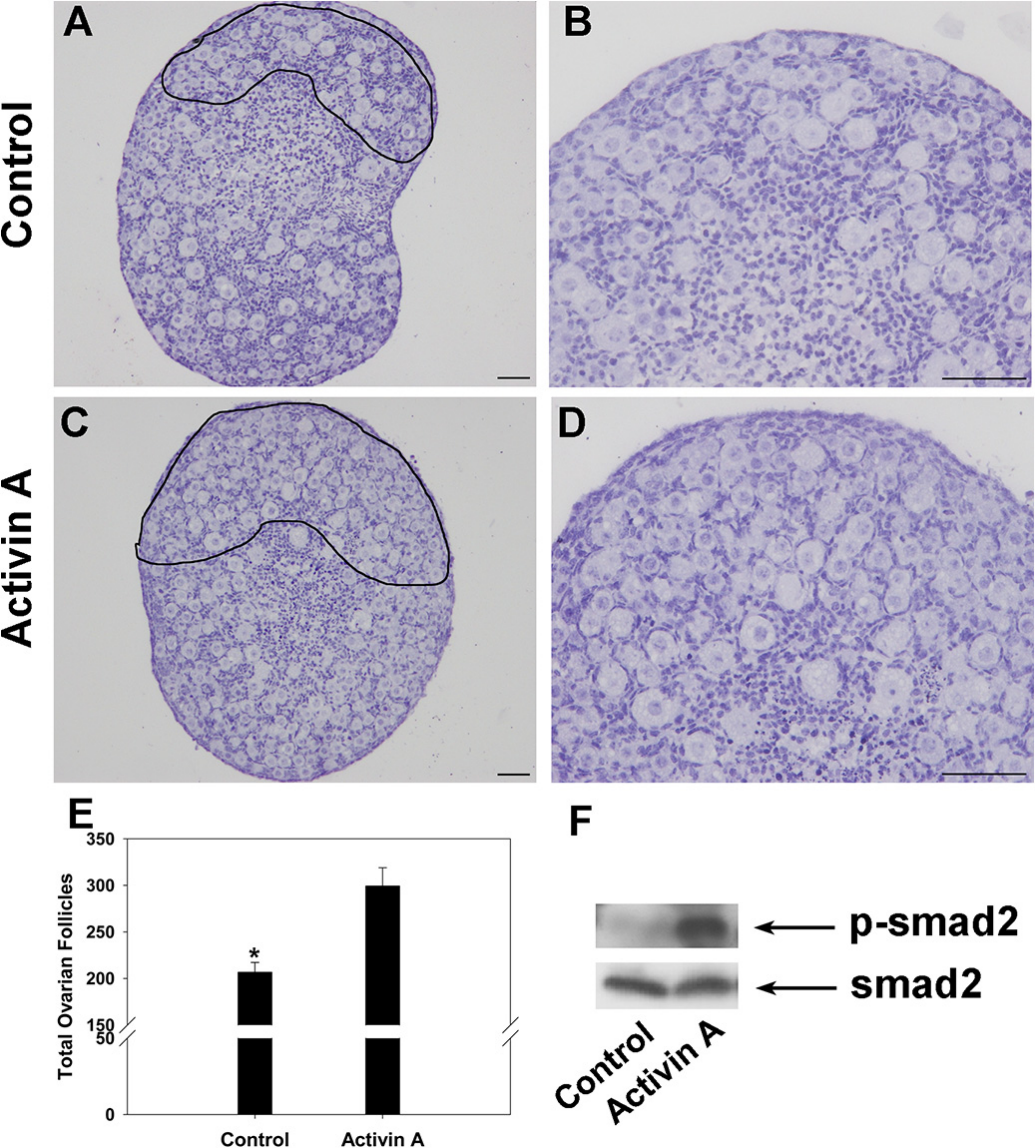
Phenotypes of ovary cultures treated with ACT A. Ovaries at 17.5 dpc were cultured for 7 days without treatment (as a control) or with 100 ng/mL ACT A. Compared to control ovaries (A, black boundary enlarged in B), there were significantly more primordial follicles in the periphery of ovaries treated with ACT A (C, black boundary enlarged in D). The total number of ovarian follicles in the largest cross-section of control and ACT A-treated ovaries were quantified (E). Western blot analysis revealed an obvious increase of the p-smad2 level in Activin A-treated ovaries relative to control group (F). Scale bars: 40 μm (A-D). Smad2 was measured as internal control. Significant differences are indicated by * (*P* < 0.05).

Since FST acts by binding and neutralizing activin, we examined the effect of FST-binding protein activin A on folliculogenesis. Ovaries at 17.5 dpc were cultured alone as a control or with 100 ng/mL ACT A for 7 days. Compared to control ovaries (Fig 7A and 7B), there were significantly more primordial follicles in the peripheral area of ovaries treated with ACT A (Fig 7C and 7D). The primordial follicles in ACT A-treated ovaries were distributed throughout the entire ovary, including the cortical area and medullary area (Fig 7C and 7D). In addition, the total number of ovarian follicles was significantly increased in ACT A-treated ovaries as compared to control ovaries (Fig 7E). To examine the effect of ACT A on cultured ovaries, we detected the phosphorylation level of smad2 in the ovaries. Western blot analysis showed that the level of p-smad2 was significantly increased in ACT A-treated ovaries as compared with controls (Fig 7F).

## Discussion

The pool of resting primordial follicles represents the entire reproductive potential of mammalian females and is established immediately after birth in mice [1]. Therefore, factors that can influence the size or quality of this pool can have significant impact on reproductive fertility. Aberrations in cyst breakdown during primordial follicle assembly and excessive depletion of the primordial follicle pool will result in ovarian diseases [28]. In humans, one of the potential causes of premature ovarian failure (POF), which includes irregular cycles, accelerated loss of primordial follicles, and early termination of ovarian activity, is accelerated depletion of the resting primordial follicle pool. Hence, understanding the molecular mechanisms driving the assembly of primordial follicles is critical for better understanding ovarian diseases and the development of therapeutic applications. Our results suggest that ACT significantly promotes primordial follicle formation while FST288 inhibits germ cell nest breakdown and primordial follicle formation via inhibition of somatic cell proliferation during folliculogenesis.

Our results regarding the pattern of expression of FST in the mouse ovary are consistent with previous studies. Previous studies have demonstrated that FST was expressed within nuclei of some but not all oocytes within nests and even in more mature follicles [23]. Besides, the predominant staining of FST was in nuclei of spermatocytes and spermatids in the seminiferous tubules in the testis [29]. In this study, weak FST staining was observed in the germ cell cytoplasm and strong staining in many germ cell nuclei but not all in 17.5 dpc, 19.5 dpc and 1 dpp mouse ovaries in our experiments, and FST expression can also be detected in granulosa cells beginning at 4 dpp. When FST288 is the only FST isoform expressed (i.e., when *FST303* and *FST315* are deleted), FST is mainly present in the cytoplasm of oocytes in adult ovaries while in neonatal ovaries many germ cells retain FST nuclear staining [23, 30]. At present, no direct articles reported that what physiological significance is conferred by this nuclear FST at the time of primordial follicle formation. However, FST was recently localized to the nucleolus in HeLa cells where it reduced ribosomal RNA synthesis and promoted survival of these cells in low glucose environments [31]. Here we found that FST was highly expressed when germ cells remained in nests on 17.5 dpc through 19.5 dpc, indicating that high FST expression within the oocyte may occur when the germ cells are still in cysts. After birth, FST expression in the ovary decreased gradually from 1 to 4 dpp, during which time large amounts of primordial follicles assemble. Although FST expression increased in 7 dpp ovaries, some of the protein may have been produced by the granulosa cells. These results indicate that FST may play different roles in oocytes and granulosa cells during the breakdown of cysts and formation of primordial follicles.

Primordial follicle production was increased by administration of ACT *in vitro* in our study. These results are consisted with a previous *in vivo* study, which found that consecutive injection of ACT for 4 days into newborn female mice resulted in an increased primordial follicle pool by promoting germ cell and somatic cell proliferation [5]. Since FST is a negative regulator of ACT, the level of FST within the ovary may significantly affect the impact of ACT. In this study, we demonstrated that administration of the FST isoform FST288 inhibited germ cell nest breakdown and primordial follicle formation by inhibiting somatic cell proliferation in cultured mouse ovaries. Our results are supported previous studies on mice lacking the other two isoforms of FST, *FST303* and *FST315*. These mice are born with increased germ cell number at birth, extended germ cell nest breakdown, and reduced germ cell apoptosis, which together contribute to an enlarged primordial follicle pool [23]. However, FST288-only mice have fertility defects including rapid depletion of primordial follicle pool, few morphologically healthy antral follicles, irregular cycles, reduced litter size, and early cessation of reproduction in later reproductive life. As a result, they are subfertile and share a number of features with human premature ovarian failure (POF) [28, 30]. In addition, FST288 is superior to FST303 and FST315 in conjugating with and inhibiting ACT [24]. Taken together, these results suggest that the three isoforms of FST have different roles in folliculogenesis.

Since there is dramatically less proliferation of granulosa cells near the ovarian cortex in FST288-treated ovaries and there are more primordial follicles in the ovaries in ACT-treated ovaries, it is likely that FST288 prevents germ cell nest breakdown and primordial follicle formation via inhibition of somatic cell proliferation. Due to a decrease in the number of somatic cells circling the cysts, they are unable to encapsulate individual germ cells. This is supported by the demonstration that overexpression of mouse FST results in an early block in folliculogenesis before antral follicle formation [32].

The primordial follicle assembly process involves programmed cell death of oocytes [33]. In ovaries without *FST303* and *FST315*, apoptosis is significantly reduced early in the breakdown process [23]. However, no significant increase in oocyte apoptosis has been observed when neonatal mice are treated with ACT [5]. While we did not observe any alteration in apoptosis in FST288-treated ovaries after 4 days of culture, further studies evaluating shorter and longer culture periods are needed to clarify whether FST288 affects oocyte apoptosis. Nevertheless, our results suggest that FST288 plays an important role in germ cell nest breakdown, mainly impacting somatic cell proliferation. Further studies are needed to elucidate why the three isoforms of FST act differently on the two types of cells during mice cyst break down and primordial folliculogenesis.

Recent studies have demonstrated that both estradiol (E2) and progesterone (P4), and Notch signaling are critical for inhibiting nest breakdown and primordial follicle formation[6–7, 34–36]. The maternal E2 and P4 may exert their actions on prenatal ovaries since their levels drop precipitously in the fetal mouse ovaries after birth, and primordial follicle formation is initiated during this period. Notch signaling by somatic cells was recently demonstrated to induce the process of germ cell nest breakdown and primordial follicle formation [34–36]. Jagged1 knockout and Notch2 knockout ovaries contained multi-oocytic follicles, which represent a failure to resolve germ cell syncytia [35]. Female mice with conditional deletion of the Notch2 gene in somatic granulosa cells of the ovary exhibited multi-oocyte follicles which were resulted from defects in breakdown of the primordial germ-cell nests [36]. Previous results in our lab also showed that down-regulation of Jagged2 and Notch1 by RNAi (RNA interference) inhibited germ cell nest breakdown and primordial follicle formation *in vitro* [8]. Besides, interactions between Notch and the ACT signaling pathway have also been described [37–40]. FST288, as the strongest ACT-neutralizing isoform, decreased activin bioavailability that causes a reduction of granulosa cell proliferation which resulted in delayed germ cell nest breakdown. So it is necessary to clarify which cell signaling pathway takes part in the process. Therefore, we examined the protein levels of Notch1, NICD1 (active Notch1), and Hey2 in *in vitro* cultured mice ovaries and found that they are significantly reduced after 7 days of culture with FST288. However, no matter whether Notch1 receptor or Notch2 receptor was down-regulated, the downstream target of Hey2 was down-regulated, which means that supervising Hey2 expression helps to identify if Notch signaling was attenuated. Based on these, in our study, we found that Hey2 was significantly decreased in FST288 treated ovaries, indicating that Notch signaling was impaired in mice ovaries. Our results suggest that reduced Notch signaling proteins in these ovaries may contribute to the reduced number of somatic cells.

In conclusion, we have demonstrated that FST288 inhibits germ cell nest breakdown and primordial follicle formation by inhibiting somatic cell proliferation. These data provide a theoretical basis to better understand the physiological role of FST288 in germ cell nest breakdown and primordial follicle assembly in the mouse ovary.
